# TNFSF4/OX40L and IKZF1/IKAROS Genetic Variant Associations with Egyptian Juvenile Systemic Lupus Erythematosus (JSLE)

**DOI:** 10.3390/biology15060489

**Published:** 2026-03-19

**Authors:** Zeinab R. Attia, Ahmed M. Amshawee, Ahmed Flayyih Hasan, Dalia Tawfeek Hussein, Rania A. Abd El Azeem, Mohamed M. Zedan, Thuraya M. Mutawi, Nanis S. El-beltagy, Mohamed A. El Basuni

**Affiliations:** 1 Mansoura University Children’s Hospital, Faculty of Medicine, Mansoura University, Mansoura 35516, Egypt; dr.dalia.tawfick@mans.edu.eg (D.T.H.); rania11112007@gmail.com (R.A.A.E.A.); thuraya.medhat@gmail.com (T.M.M.); nanissalah@mans.edu.eg (N.S.E.-b.); mohamedelbasuni1@gmail.com (M.A.E.B.); 2 Department of Radiology, College of Health and Medical Technology, University of Hilla, Babylon 51002, Iraq; ahmed_meki@hilla-unc.edu.iq; 3 Biotechnology Research Center, Al-Nahrain University, Baghdad 64074, Iraq; ahmed_flayyih@nahrainuniv.edu.iq; 4 Department of Medical Laboratory Techniques, College of Health and Medical Technology, Al-Farabi University, Baghdad 64001, Iraq; 5 Department of Clinical Laboratory Sciences, College of Medical Applied Sciences, University of Hafr Al Batin, Hafr Al Batin 1803, Saudi Arabia; 6 Department of Pediatrics, Faculty of Medicine, Mansoura University, Mansoura 35516, Egypt; mmzedan@yahoo.com

**Keywords:** juvenile SLE, TNFSF4, IKZF1, polymorphism

## Abstract

Background: The etiopathogenesis of juvenile systemic lupus erythematosus (JSLE), a complex and complicated illness, is unknown. Genetic, environmental, and dysregulated immune system responses are all thought to contribute to the disease’s etiology. Important immunological molecules that regulate different immune cells and are associated with autoimmune disorders are TNFSF4 and IKZF1. Thus, our purpose was to discover if TNFSF4 and IKZF1 mutations left the Egyptian population genetically predisposed to SLE. Methods: Using real-time polymerase chain reaction (RT-PCR), polymorphism analysis of the TNFSF4 rs1234315 C/T and IKZF1 rs11980379 C/T genes was performed on extracted DNA from JSLE patients and healthy controls. Results: TNFSF4 frequencies (rs1234315 T allele, CT, TT genotypes, dominant and recessive models) were substantially associated with a higher incidence of JSLE (*p* < 0.05) compared to healthy controls. Conversely, IKZF1 frequencies (rs11980379 T allele, TC, TT genotypes, and dominant model) significantly correlated with a lower incidence of JSLE. Furthermore, the TC + CC rs11980379 genotype was identified as significantly associated with lower kidney biopsy grades and a lower incidence of lupus nephritis. Conclusions: Our findings suggest that TNFSF4 and IKZF1 polymorphisms affect vulnerability to juvenile SLE.

## Simple Summary

Genetic variables, immune complex deposition, complement activation, hormonal factors, and immune cell dysregulation all contribute to varying degrees to the pathogenesis of Juvenile systemic lupus erythematosus (JSLE). Important immunological molecules that regulate different immune cells and are associated with autoimmune disorders are TNFSF4 and IKZF1. Single-nucleotide polymorphisms (SNPs), the most prevalent type of genetic variation in the human genome, may influence gene function if present. In this study, we assessed the possible effects of rs11980379 C/T in the IKZF1 gene and rs1234315 C/T in the TNFSF4 gene on the susceptibility of Egyptian children to SLE. In addition, we also studied the distribution of these genetic variants and SLE clinical outcomes. This is the first study that we are aware of that assesses the relationship between these loci and the illness in our population. To examine these genetic polymorphisms, we performed a real-time polymerase chain reaction using the TaqMan^TM^ SNP genotyping assay on the Artus Rotor-Gene Qiagen equipment (QIAGEN, Hilden, Germany). The allelic and genotypic frequencies of rs1234315 and rs11980379 were shown to be substantially linked with the disease.

## 1. Introduction

Juvenile systemic lupus erythematosus (JSLE), an immune-induced condition with a variety of immunologic and clinical manifestations, combines autoimmune and autoinflammatory mechanisms [1,2]. The loss of immunological tolerance in people with systemic lupus erythematosus (SLE) promotes the formation of autoantibodies that target many organs and tissues, including the skin, joints, and kidneys. Through increased immunological responses, immune cells play critical roles in the onset and course of SLE [3]. JSLE, an unusual but severe syndrome marked by disease onset before age 18, affects 15–20% of subjects with SLE [4]. Lupus nephritis (LN) is a significant side effect of SLE that often leads to end-stage renal disease. Furthermore, the prevalence of LN varies based on racial and cultural differences worldwide [5]. The disease’s precise causes are yet unknown. Nonetheless, it has been demonstrated that the pathophysiology of SLE involves both hereditary and environmental variables [6].

Numerous studies have demonstrated that the tumor necrosis factor, a member of the superfamily 4 (TNFSF4), plays a role in tissue inflammation by regulating the synthesis of pro-inflammatory cytokines and mediators, indicating its involvement in the development of autoimmunity in both human and animal diseases [7]. The inflammatory factor TNFSF4 has been linked to several illnesses, particularly autoimmune disorders such as SLE, suggesting that TNFSF4 is vital to the pathophysiology of many conditions and that blocking TNFSF4 may prevent illness onset [8]. Numerous immune cells, including dendritic cells, macrophages, T cells, and B cells, express it [9]. Furthermore, TNFSF4 signalling is critical for immunological responses and can be responded to by several immune cells [6]. Prior research found elevated TNFSF4 expression in lupus models in mice and patients, particularly LN [6]. Overall, these findings suggest that TNFSF4 may have a role in the etiology of autoimmune disorders, particularly SLE. The TNFSF4 gene, located on chromosome 1 (1q25), encodes the cytokine OX40L (tumor necrosis factor (ligand), member of the superfamily 4) and consists of two introns and three exons [10,11]. The association between TNFSF4 rs1234315 polymorphism and SLE has been the subject of numerous studies; however, the findings presented in this investigation were inconsistent. Disparities between racial and ethnic groupings could be the cause of this.

Ikaros is a zinc finger transcription factor and a member of the Krüppel family, which is called the IKAROS family zinc finger protein family (IKZF). The Ikaros family zinc finger 1 (IKZF1) gene, located on chromosome 7p12.2 and comprising eight exons, encodes the transcription factor Ikaros, which plays a crucial role in regulating the development of the immune and lymphoid systems [12]. According to earlier research, IKZF1 mRNA expression levels substantially dropped in SLE patients, indicating that IKZF1 plays a crucial role in the distinctive SLE cascade [13]. IKZF1 mutations or abnormal expressions are strongly linked with the onset and course of a variety of immune-related disorders, including autoimmune illnesses [14]. In the meantime, many single-nucleotide polymorphisms (SNPs), including rs11980379 and rs4132601, have also been found to be susceptibility variations for several immunological illnesses, including SLE, Crohn’s disease, inflammatory bowel disease (IBD), and primary Sjoègren syndrome [15].

To put it simply, an abundance of evidence from multiple studies is emerging that supports the likely involvement of TNFSF4 and IKZF1 in the development of SLE. There is currently no proof linking the rs1234315 and rs11980379 SNPs to the severity of SLE in Egyptian children and adolescents. The most prevalent type of genetic variation in the human genome, SNPs, if present, can change a gene’s function [16]. An increased risk of autoimmune diseases, such as SLE, has been associated with variants in these genes [15,17]. We designed the current study to determine the gene polymorphisms of TNFSF4 and IKZF1 in JSLE patients and investigate whether these SNPs are associated with the risk of developing SLE in those patients.

## 2. Materials and Methods

### 2.1. Ethical Statement Approval

The ethical committee board [IRB #: R.23.01.2012] at the Faculty of Medicine, Mansoura University, Egypt, approved this work. Additionally, the Helsinki Guidelines’ declaration procedures were followed when the study process started. To be eligible to participate in this study, all recruited subjects were required to give their informed written consent.

### 2.2. Study Participants

This retrospective case–control study included 232 participants: 110 SLE patients (12.7% male, and 87.3% female) and 122 unrelated autoimmune disease-free controls (15.6% male, and 84.4% female) who were similar in age, gender, and geographic location. All SLE patients were recruited from rheumatic disease outpatient clinics at Mansoura University Children’s Hospital, Faculty of Medicine, Mansoura, Egypt. The severity of the disease was assessed using the Systemic Lupus International Collaborating Clinics/American College of Rheumatology (SLICC/ACR) damage index (SDI) [18]. This study did not include SLE individuals with a history of cancer, other autoimmune disorders, hepatic troubles, renal diseases, diabetes mellitus, metabolic diseases, or other endocrine abnormalities. In addition to renal biopsy grades of SLE cases with nephritis based on the International Society of Nephrology/Renal Pathology Society (ISN/RPS) 2003 classification of lupus nephritis. All 110 patients underwent renal biopsy; 87 had biopsy-confirmed lupus nephritis, while 23 had biopsy-proven non-LN renal pathology. Information on clinical manifestations and demographics was gathered from medical records stored in the Medical Archive Service and Health Information.

### 2.3. Sample Collection

Under rigorous aseptic conditions, 5 mL of venous blood was drawn from each participant in this study. The blood samples were divided into two aliquots. For hematological and genetic purposes, the first portion was collected in a Vacutainer tube containing EDTA (ethylene diamine tetra-acetic acid), an anticoagulant; for biochemical and serological measurements, the second portion was processed in serum separator tubes, and a serum aliquot was separated by centrifugation. Additionally, the DIALAB biochemical instrument (DIALAB GmbH, Wiener Neudorf, Vienna, Austria) was used to perform biochemical assessments, and the ELISA technique (INOVA Diagnostic, San Diego, CA, USA) was employed to evaluate Anti-Nuclear Antibodies (ANA) and Anti-ds-DNA tests. Furthermore, the Abbott CELL-DYN 3700 SL hematology instrument (Abbott Diagnostics, Chicago, IL, USA) was used to validate the hematological parameter estimates.

### 2.4. Purification and Extraction of Genomic DNA

In accordance with the manufacturer’s instructions, a handy spin column built exclusively on silica-based membrane technology was used to extract genomic DNA from entire blood samples (Gene JET, Thermo Fisher Scientific, Waltham, MA, USA). Additionally, we used the Nanodrop ND-1000 Spectrophotometer (Nanodrop Technologies, Wilmington, NC, USA) to confirm the extracted genomic DNA [19]. Until the genetic amplification procedure, all the collected samples were maintained at a temperature below 80 °C.

### 2.5. TNFSF4 rs1234315 C/T and IKZF1 rs11980379 C/T Variant Amplification

Genotyping for rs1234315 C/T in the TNFSF4 and rs11980379 C/T in the IKZF1 genes was performed using Custom TaqMan^TM^ SNP (Foster City, CA, USA) genotyping assays in which a fluorogenic probe, consisting of an oligonucleotide labelled with both a fluorescent reporter dye (FAM or VIC) and a quencher dye, is included in a typical PCR. Amplification of the probe-specific product induces probe fragmentation, thereby increasing reporter fluorescence [20,21]. Each primer and probe set was used in the TaqMan SNP genotyping assay {(Assay ID: C___8920846_10 for rs1234315 C/T, C____199416_10 for rs11980379 C/T), CatLog number: 4351379} via the Artus Rotor-Gene Qiagen fast real-time (RT) PCR System (software 2.1.0; Applied Biosystems, Foster City, CA, USA), in accordance with the information on the Applied Biosystems website (http://www.appliedbiosystems.com, accessed on 22 February 2023).

The PCR was performed according to the manufacturer’s instructions provided by Applied Biosystems (San Diego, CA, USA). The PCR thermal cycling was as follows: A final volume of 20 μL was used for each PCR reaction, which contained 10 μL of TaqMan^TM^ Genotyping Master Mix (Thermo Fisher Scientific, San Diego, CA, USA) and 20 ng of genomic DNA. The PCR reaction started at 95 °C for 10 min and was repeated 45 times at 95 °C for 15 s. Multiple positive (Centre d’Etude du Polymorphisme Humain (CEPH)) and negative controls were used in each genotyping assay, and allele calls were verified with HapMap data for validation. To rule out genotyping errors, 50% of the original specimens were randomly evaluated for replication. There were no significant differences between genotypes determined in duplicate.

### 2.6. Statistical Analysis

The Statistical Package for the Social Sciences (IBM Corp., 2017, Armonk, NY, USA) was used to analyze the gathered data—version 25.0 of IBM SPSS Statistics for Windows. The link between two qualitative variables was investigated using the chi-square test. Fisher’s exact test was used to evaluate the relationship between two qualitative variables when the expected count was less than five in more than 20% of cells. The ANOVA Test was used to assess the statistical significance of the difference in parametric variables between the two research teams. The statistical significance of the difference in a non-linear variable between two study groups was assessed using the Mann–Whitney U test. The Kruskal–Wallis Test was used to assess the statistical significance of a non-parametric factor’s difference between more than two studies. When the dependent variable was categorical, risk variables were predicted using binary logistic regression. Risk variables were predicted using binary logistic regression analysis when the dependent variable was categorical. A measure of the relationship between exposure and an outcome is called an odds ratio (OR). The odds of an event occurring given a specific exposure, in contrast to the odds of the result occurring in the absence of that exposure, are represented by the OR. OR = 1: The odds of an outcome are unaffected by exposure. Exposure is associated with higher odds (risk) of an event if OR > 1; OR < 1: Reduced odds are associated with exposure (protective). To determine the precision of the OR, the 95% confidence interval (CI) is employed. A large CI implies that the OR is highly precise, whereas a modest CI indicates low precision. A *p*-value is deemed significant if it is less than 0.05 at a 95% confidence interval.

## 3. Results

### 3.1. Characteristics of the Studied Participants

The 232 participants in this study were divided into two subgroups: 110 SLE patients with a mean age of 12.7 ± 3.17 years and 122 healthy controls from the same area with a mean age of 12.9 ± 2.63 years. SLE patients and control volunteers did not show significant differences in terms of age or gender (*p* > 0.05; Table 1). White blood cell (WBC) counts, platelet counts, hemoglobin, Ca, P, C3, and C4 complement levels were significantly decreased in SLE patients, and elevated creatinine levels were observed (Table 1). While 96.4% and 92.7% of SLE participants had positive ANA and ds-DNA, respectively, all control subjects (100%) had negative ANA and ds-DNA.

Additionally, Table 1 shows that SLE patients exhibit a range of clinical symptoms. These symptoms have a considerably different prevalence rate. Vasculitis and fever are examples of low-prevalence symptoms that occurred in 5–15% of SLE cases. Oral ulcers, neurological symptoms, alopecia, photosensitivity, and arthritis were among the symptoms with a medium rate of prevalence, occurring in 25–48% of cases. High-frequency symptoms, such as immunologic disorders, kidney disease, and malar rash, were present in 65–100% of cases.

### 3.2. The Allelic and Genotypic Frequencies of the TNFSF4 and IKZF1 Variants with SLE

The genotype and allele frequencies of the examined SNPs in the case and control groups are listed in Table 2. As indicated in Table 2 (*p*-value > 0.05), the genotype frequencies of TNFSF4 rs1234315 C/T and IKZF1 rs11980379 C/T in controls or cases adhered to the Hardy–Weinberg equilibrium (HWE). This suggests that the information is trustworthy and typical of genetic association analysis.

We examined the variations in genotype and allele frequency distributions between patients and controls as in Table 2, the genotypes “CT”, “TT” and “T” allele in rs1234315 were associated with increased risk of SLE [(*p* = 0.044, OR = 1.48, 95% CI = 1.01–2.17), (*p* = 0.004, OR = 1.95, 95% CI = 1.24–3.08), and (*p* = 0.003, OR = 1.42, 95% CI = 1.13–1.79)], respectively. Additionally, we used logistic regression modelling in SNP Stats to evaluate the relationship between this SNP and SLE risk, utilizing dominant and recessive genetic models. As shown in Table 2, crude analysis revealed that the genotypes “CT + TT” and “TT” in rs1234315 were associated with increased risk of SLE under the dominant model (*p* = 0.008, OR = 1.62, 95% CI = 1.13–2.32), and under the recessive model (*p* = 0.032, OR = 1.53, 95% CI = 1.04–2.26), respectively. The recessive model for IKZF1 rs11980379 was not statistically significant, likely due to the low frequency of CC homozygotes, suggesting that the observed genetic association is more consistent with dominant than recessive inheritance.

Comparing patient groups to controls, as illustrated in Table 2, the genotype distributions of the IKZF1 rs11980379 C/T gene polymorphism revealed that the “TC”, “CC” genotypes, “C” allele and Combined model “TC + CC” were associated with reduced risk of SLE [(*p* < 0.001, OR = 0.51, 95% CI = 0.36–0.72), (*p* = 0.045, OR = 0.43, 95% CI = 0.19–0.98), (*p* < 0.001, OR = 0.59, 95% CI = 0.45–0.78), and (*p* < 0.001, OR = 0.50, 95% CI = 0.36–0.70], respectively.

### 3.3. The Impact of TNFSF4 rs1234315 C/T and IKZF1 rs11980379 C/T Variants with Clinical/Laboratory Features in SLE Patients

As illustrated in Table 3, the genotypes of TNFSF4 rs1234315 and IKZF1 rs11980379 are not associated with duration, SLEDAI, ANA, dsDNA, urine analysis, and renal biopsy in SLE patients. For TNFSF4 rs1234315, significant differences in C3 levels were observed among the CC, CT, and TT genotypes, with the highest levels found in the CC genotype among SLE patients (*p* = 0.032; Table 3). Although Fisher’s exact test demonstrated a statistically significant association, the CC genotype subgroup is very small; therefore, this finding should be interpreted cautiously and considered exploratory rather than definitive. Consanguinity and the presence of a similar condition exhibited no significant relationships with either genotype. Regarding lupus nephritis (LN), the presence of the CC genotype of IKZF1 rs11980379 was significantly associated with protection against its occurrence, as illustrated in Table 4 (*p* = 0.002). However, Fever, oral ulcers, vasculitis, arthritis, malar rash, photosensitivity, alopecia, and neurological manifestations did not exhibit significant associations with either genotype (*p* > 0.05; Table 4).

Furthermore, the dominant models for both TNFSF4 rs1234315 and IKZF1 rs11980379 did not show significant associations with SLEDAI scores. However, the presence of the “TC + CC” genotype of IKZF1 rs11980379 was significantly associated with lower grades of renal biopsy, as well as a lower likelihood of lupus nephritis [(*p* = 0.015, OR = 0.69, 95% CI = 0.51–0.93), (*p* = 0.016, OR = 0.49, 95% CI = 0.27–0.88)], respectively. The observed association between lower grades on renal biopsy and the dominant model (TC + CC) can only be explained by the TC genotype, as no CC carriers underwent renal biopsy, indicating that CC did not contribute to the regression model. However, as shown in Table 5, other clinical indicators did not exhibit significant correlations with either genotype in these regression models.

### 3.4. Comparative Analysis of TNFSF4 rs1234315 C/T and IKZF1 rs11980379 C/T Genes in SLE

Table 6 summarizes a comparative analysis of our findings with existing international reports (including both consistent and inconsistent studies) on the association between the TNFSF4 rs1234315 and IKZF1 rs11980379 SNPs and SLE.

## 4. Discussion

Genetic variables, immune complex deposition, complement activation, hormonal factors, and immune cell dysregulation are all involved to varying degrees in the pathogenesis of JSLE; this suggests that future patient categorization based on immunological phenotypes may be achievable [26]. Immunologic deficiencies, blood cell abnormalities, arthritis, kidney diseases, and skin disease are some of the clinical manifestations of SLE, a multifactorial autoimmune illness. According to our research, the most common conditions in SLE cases are immunological, hematological, renal, and cutaneous problems.

In recent decades, numerous genetic factors associated with SLE have been identified. Meanwhile, the incidence and prevalence of SLE vary significantly within various ethnic or geographic groups, suggesting that SLE risk is influenced by genetic variability [22,27]. Thus, it is essential to investigate the genes and loci associated with SLE susceptibility in young Egyptian populations. Gene polymorphism is crucial in understanding illness susceptibility and the variety of clinical disease presentations. The most prevalent variant in a population’s DNA sequence is called an SNP. Numerous studies have demonstrated the critical role that SNPs play in the development of SLE [28]. In this study, we assessed the possible effects of rs11980379 C/T in the IKZF1 gene and rs1234315 C/T in the TNFSF4 gene on the susceptibility of Egyptian children to SLE. This is the first study that we are aware of that assesses the relationship between these loci and the illness in our population. Both the allelic and genotypic frequencies of rs1234315 and rs11980379 have been identified to be substantially linked with the disease.

The inflammatory factor TNFSF4 has been linked to multiple diseases, particularly autoimmune disorders, suggesting that TNFSF4 is essential to the pathophysiology of many conditions and that blocking TNFSF4 may prevent disease onset [8]. However, the results have not been consistent across studies, and comparable research has not been conducted on our population. The SNP rs1234315, a T/C variant located approximately 2 kb away from the 5′ UTR of the TNFSF4 gene [23], was selected for the current investigation. TNFSF4 genetic variants have been studied in relation to risk and clinical outcomes in several autoimmune diseases, including SLE [24]. These polymorphisms have been identified as probable causes of SLE.

Our results showed that TNFSF4 rs1234315 C/T is a risk factor for SLE, with significant differences in rs1234315 genotype and allele frequencies between SLE patients and controls in both dominant and recessive models. This finding supports a relationship (*p* < 0.05) between the TNFSF4 rs1234315 C/T genotype and the severity of SLE in Egyptian juveniles. This suggests that TNFSF4 may be a common genetic determinant for SLE onset. The stratification analyses showed that rs1234315 was more strongly associated with SLE patients with arthritis [25]. Similarly, a meta-analysis conducted by Wang et al. [6] validated this association across cohorts of Hispanics, Europeans, East Asians, and Chinese populations from Hong Kong. This is consistent with the results of Xu et al. [24], who observed that among Guangxi Chinese groups, this polymorphism was associated with SLE. The adhesion of activated T cells to vascular endothelial cells, which can lead to B cell activation and differentiation, is regulated by the TNFSF4 gene, according to biology. This implies that the pathophysiology of SLE may include TNFSF4 [25]. All the previously cited data suggest that TNFSF4 may have a role in the onset of SLE. However, our findings were contradicted by another investigation. Likewise, in Malaysian, Indian, and other Chinese ethnicities, Chua et al.’s [17] study found no evidence of the lupus link at this locus. Population variability may explain the discrepancies in outcomes between our study and these investigations.

Although there is substantial evidence that IKZF1 genes play a part in SLE, it remains elusive how these genes interact with the immunological pathogenesis and clinical manifestations of SLE. One SNP, rs11980379 C/T in IKZF1, was found in this investigation to be strongly linked to SLE susceptibility in Egyptian children and adolescents. According to the results, the TC, CC genotypes, C allele, and the Combined model TC + CC of the rs11980379 SNP were correlated with reduced susceptibility to SLE. To the best of our knowledge, no study has shown a link between SLE in Egyptian children and adolescents and the IKZF1 rs11980379 SNP. Complementary to this, numerous earlier studies have found that certain IKZF1 SNPs are primarily associated with autoimmune conditions such as Crohn’s disease, inflammatory bowel disease, and primary Sjoègren syndrome [29,30,31]. According to the study by Chen et al. [15], who discovered that statistical differences between SLE patients and healthy people in genotype distributions of IKZF1 rs11980379 (dominant model, OR = 0.67, 95% CI = 0.50–0.89, *p* = 0.006; recessive model, OR = 0.48, 95% CI = 0.26–0.91, *p* = 0.02; allelic model, OR = 0.65, 95% CI = 0.50–0.84, *p* < 0.001). This suggests that the IKZF1 rs11980379 SNP was associated with reduced risk (protective) of SLE in adult Chinese populations, a finding consistent with our results. IKZF1 is one of the SLE susceptibility genes; thus, it may be a candidate SLE gene in Egyptian children and adolescents, supporting the idea that IKZF1 is associated with a variety of autoimmune disorders. Findings related to low-frequency genotypes, particularly the IKZF1 rs11980379 CC genotype, should be interpreted cautiously due to limited statistical power and require validation in a larger, multicenter cohort.

Regression analysis revealed that SLE patients with C3 may be associated with TNFSF4 rs1234315 (*p* = 0.032). Although the CC genotype was less frequent among patients, its association with higher C3 levels suggests a protective immunological profile. This supports a potential modulatory role in disease biology. In addition, our findings demonstrated a genetic correlation between IKZF1 rs11980379 and lupus nephritis (*p* = 0.002), and further research is required to investigate the precise mechanism. Meanwhile, we were unable to find any meaningful correlation between other clinical and laboratory studies of SLE and the polymorphisms of rs1234315 C/T within TNFSF4 or rs11980379 C/T in IKZF1.

Differences in association direction across studies may reflect population-specific LD structures, allele frequency distributions, gene–gene interactions, and environmental modifiers, rather than true biological contradictions. North African/Middle Eastern populations remain underrepresented in SLE genetic studies. In addition, Egyptian genetic structure is admixed (African–Mediterranean–Middle Eastern ancestry), which may influence SNP–disease associations. This may explain both the variation in effect sizes and the direction of associations. Although IKZF1 has been repeatedly implicated in GWAS of SLE susceptibility, rs11980379 specifically remains under-investigated, which explains the limited number of direct comparison studies available for this variant. This study is based on genetic association analysis and does not provide functional validation. The identified SNPs may represent markers in linkage disequilibrium with causal variants, rather than being directly functional themselves. Functional genomics studies are required to determine their biological effects on gene expression, immune regulation, and disease pathogenesis.

However, this study may have a few drawbacks. Primarily, the population and number of patients in this single-center study are small. Secondly, this study focused on the associations between TNFSF4 and IKZF1 polymorphisms and susceptibility to SLE, as well as clinical symptoms of SLE, and did not include experimental confirmation of the biological activities underlying the SNPs, which is an essential field for future research. Finally, each gene has just one genetic variation examined. So, these results are exploratory and hypothesis-generating rather than definitive. Further research with larger sample sizes, fine-mapping studies of more IKZF1 and TNFSF4 variants, and a range of ethnic groups is required to validate the results of this study. Despite these drawbacks, our research found that two TNFSF4 and IKZF1 variants (rs1234315 and rs11980379) are linked to SLE susceptibility in Egyptian children and adolescents. Additionally, the SNP IKZF1 rs11980379 has been identified to have been associated with lower renal biopsy grades and a decreased risk of lupus nephritis, which likely plays a role in the disease’s progression.

## 5. Conclusions

In conclusion, our research independently verifies and suggests the robust correlation between SLE susceptibility in Egyptian pediatric populations and SNPs rs1234315 in TNFSF4 and rs11980379 in IKZF1. It suggests that various populations share some genetic characteristics; however, the significance of gene heterogeneity may not be disregarded. Investigating the complete genetic basis of SLE in different cultures may contribute to a better understanding of the disease’s pathophysiology.

## Figures and Tables

**Table 1:** Demographic, Laboratory, and Clinical Data among SLE patients and Controls.

Parameters		SLE (*n* = 110)	Control (*n* = 122)	*p*
Sex: *n* (%)	Male	14 (12.7%)	19 (15.6%)	0.535
	Female	96 (87.3%)	103 (84.4%)	
Age: Mean ± SD		12.7 ± 3.17	12.9 ± 2.63	0.776
WBCs (×10^12^/L): Mean ± SD		4.83 ± 1.06	6.68 ± 1.47	<0.001 *
RBCs (×10^12^/L): Mean ± SD		4.25 ± 0.56	4.96 ± 0.54	<0.001 *
Hemoglobin (g/dL): Mean ± SD		11 ± 1.41	12.2 ± 0.82	<0.001 *
Platelets (×10^9^/L): Mean ± SD		251 ± 84.2	324 ± 66	<0.001 *
S. Phosphorous: Mean ± SD		4.83 ± 0.54	5.07 ± 0.36	<0.001 *
S. Calcium (mg/dL): Mean ± SD		8.87 ± 0.58	9.22 ± 0.37	<0.001 *
S. Creatinine (mg/dL): Mean ± SD		0.86 ± 0.29	0.5 ± 0.11	<0.001 *
C3: Mean ± SD		92.6 ± 17.2	119 ± 14.5	<0.001 *
C4: Mean ± SD		12.2 ± 5.97	20.9 ± 5.38	<0.001 *
ANA: *n* (%)		106 (96.4%)	-	-
Anti-ds DNA: *n* (%)		102 (92.7%)	-	-
Urine analysis: *n* (%)				
Protein	Nil	60 (54.5%)	-	-
	1	26 (23.6%)	-	-
	2	9 (8.2%)	-	-
	3	15 (13.6%)	-	-
Casts	Hyaline	11 (10.0%)	-	-
	Granular	8 (7.3%)	-	-
Clinical manifestations				
Consanguinity: *n* (%)		41 (37.3%)	-	-
Similar condition: *n* (%)		28 (25.5%)	-	-
Duration (years): Median (Min.—Max.)		3 (0.5–10)	-	-
SLEDAI: Median (Min.—Max.)		9 (0–21)	-	-
Lupus nephritis, Renal biopsy (*n* = 87): *n* (%)	1	8 (9.2%)	-	-
	2	13 (14.9%)	-	-
	3	37 (42.5%)	-	-
	4	24 (27.6%)	-	-
	5	5 (5.7%)	-	-
Fever: *n* (%)		15 (13.6%)	-	-
Oral ulcer: *n* (%)		25 (22.7%)	-	-
Vasculitis: *n* (%)		5 (4.5%)	-	-
Arthritis: *n* (%)		48 (43.6%)	-	-
Malar rash: *n* (%)		65 (59.1%)	-	-
Photosensitivity: *n* (%)		45 (40.9%)	-	-
Alopecia: *n* (%)		39 (35.5%)	-	-
Neurological: *n* (%)		27 (24.5%)	-	-

Values are expressed as mean ± SD, *n* (%), Median (Min.—Max.). SLE: systemic lupus erythematosus; SLEDAI: systemic lupus erythematosus Disease Activity Index; ANA: anti-nuclear Antibodies, Anti-ds-DNA: anti-double-stranded DNA; WBCs: white blood cells; C3 & C4: complement components; S: serum; *n*: number in each group, SD: Standard deviation; Min.: Minimum; Max.: Maximum; * *p* < 0.05 versus the control group is considered statistically significant. Tests: X2: Chi-Square test; U: Mann–Whitney. Non-numerical data were expressed by using *n* (%).

**Table 2:** Genotype distributions among SLE patients compared to controls.

Parameters	Model	Genotype	SLE (*n* = 110)	Control (*n* = 122)	*p*	OR (95% CI)
TNFSF4 rs1234315 C/T	Genotypes	CC	24 (21.8%)	46 (37.7%)		Reference
		CT	54 (49.1%)	55 (45.1%)	0.044 *	1.48 (1.01–2.17)
		TT	32 (29.1%)	21 (17.2%)	0.004 *	1.95 (1.24–3.08)
	Dominant	CC	24 (21.8%)	46 (37.7%)		Reference
		CT + TT	86 (78.2%)	76 (62.3%)	0.008 *	1.62 (1.13–2.32)
	Recessive	CC + CT	78 (70.9%)	101 (82.8%)		Reference
		TT	32 (29.1%)	21 (17.2%)	0.032 *	1.53 (1.04–2.26)
	Allele	C	102 (46.4%)	147 (60.2%)		Reference
		T	118 (53.6%)	97 (39.8%)	0.003 *	1.42 (1.13–1.79)
HWE			0.892	0.516		
IKZF1 rs11980379 C/T	Genotypes	TT	76 (69.1%)	52 (42.6%)		Reference
		TC	31 (28.2%)	62 (50.8%)	<0.001 *	0.51 (0.36–0.72)
		CC	3 (2.7%)	8 (6.6%)	0.045 *	0.43 (0.19–0.98)
	Dominant	TT	76 (69.1%)	52 (42.6%)		Reference
		TC + CC	34 (30.9%)	70 (57.4%)	<0.001 *	0.50 (0.36–0.70)
	Recessive	TT + TC	107 (97.3%)	114 (93.4%)		Reference
		CC	3 (2.7%)	8 (6.6%)	0.171	0.57 (0.25–1.28)
	Allele	T	183 (83.2%)	166 (68.0%)		Reference
		C	37 (16.8%)	78 (32.0%)	<0.001 *	0.59 (0.45–0.78)
HWE			0.940	0.063		

Values are expressed as percentages; * *p* < 0.05 is considered statistically significant. *n*: number in each group, SLE: systemic lupus erythematosus, TNFSF4: Tumor necrosis factor superfamily member 4, IKZF1: IKAROS family zinc finger 1. OR: odds ratio; CI, confidence interval; HWE, Hardy–Weinberg equilibrium (HWE). Test: Logistic regression analysis.

**Table 3:** Association of TNFSF4 rs1234315 C/T, and IKZF1 rs11980379 C/T Genotypes with SLE Laboratory Features.

Parameters		TNFSF4 rs1234315 C/T			*p*	IKZF1 rs11980379 C/T			*p*
		CC (*n* = 24)	CT (*n* = 54)	TT (*n* = 32)		TT (*n* = 76)	TC (*n* = 31)	CC (*n* = 3)	
S. Creatinine		0.78 ± 0.26	0.92 ± 0.32	0.84 ± 0.25	0.124	0.88 ± 0.3	0.85 ± 0.28	0.67 ± 0.12	0.408
S. Phosphate		4.84 ± 0.48	4.84 ± 0.57	4.81 ± 0.56	0.977	4.87 ± 0.52	4.77 ± 0.59	4.47 ± 0.57	0.391
S. Calcium		8.92 ± 0.56	8.89 ± 0.55	8.8 ± 0.64	0.696	8.85 ± 0.57	8.98 ± 0.57	8.37 ± 0.64	0.171
WBCs		5.04 ± 0.97	4.7 ± 0.99	4.9 ± 1.24	0.396	4.92 ± 1.11	4.66 ± 0.98	4.37 ± 0.15	0.396
RBCs		4.24 ± 0.51	4.16 ± 0.59	4.39 ± 0.52	0.152	4.23 ± 0.55	4.31 ± 0.58	4.07 ± 0.59	0.649
Hemoglobin		11 ± 1.49	10.94 ± 1.41	11.2 ± 1.38	0.666	11.1 ± 1.45	11 ± 1.37	10.3 ± 0.87	0.637
Platelets		257 ± 87.3	243 ± 84.2	262 ± 83.1	0.644	244 ± 82.7	261 ± 86.9	340 ± 40.8	0.099
C3		99 ± 14.8	90.6 ± 16.8	91.2 ± 18.7	0.032 *	93.4 ± 17	92.1 ± 16.8	76.3 ± 24.5	0.308
C4		13 ± 5.71	11.3 ± 5.54	13.1 ± 6.77	0.236	11.7 ± 5.75	13.5 ± 6.27	9.67 ± 8.08	0.335
Anti-ds DNA		(*n* = 22)	(*n* = 52)	(*n* = 28)		(*n* = 69)	(*n* = 30)	(*n* = 3)	
		474 ± 251	490 ± 212	399 ± 212	0.068	456 ± 215	470 ± 236	500 ± 336	0.985
ANA		(*n* = 23)	(*n* = 54)	(*n* = 29)		(*n* = 73)	(*n* = 30)	(*n* = 3)	
		124 ± 55.7	143 ± 66.9	117 ± 45.1	0.138	124 ± 48.3	148 ± 82.2	151 ± 17.4	0.270
Urine analysis									
Protein	NIL	13 (54.2%)	27 (50.0%)	20 (62.5%)	0.810	40 (52.6%)	17 (54.8%)	3 (100%)	0.795
	1	6 (25.0%)	13 (24.1%)	7 (21.9%)		20 (26.3%)	6 (19.4%)	0 (0%)	
	2	3 (12.5%)	4 (7.4%)	2 (6.3%)		5 (6.6%)	4 (12.9%)	0 (0%)	
	3	2 (8.3%)	10 (18.5%)	3 (9.4%)		11 (14.5%)	4 (12.9%)	0 (0%)	
Cast	Absent	22 (91.7%)	43 (79.6%)	26 (81.3%)	0.416	63 (82.9%)	26 (83.9%)	2 (66.7%)	0.599
	Present	2 (8.3%)	11 (20.4%)	6 (18.8%)		13(17.1%)	5 (16.1%)	1 (33.3%)	
Renal biopsy		(*n* = 20)	(*n* = 44)	(*n* = 23)		(*n* = 65)	(*n* = 22)	(*n* = 0)	
	1	3 (15%)	4 (9.1%)	1(4.3%)	0.352	3 (4.6%)	5 (22.7%)		0.110
	2	1 (5%)	8 (18.2%)	4 (17.4%)		9 (13.8%)	4 (18.2%)		
	3	12 (60%)	15 (34.1%)	10 (43.5%)		29 (44.6%)	8 (36.4%)		
	4	3 (15%)	13 (29.5%)	8 (34.8%)		19 (29.2%)	5 (22.7%)		
	5	1 (5%)	4 (9.1%)	0 (0%)		5 (7.7%)	0 (0%)		

Values are expressed as percentages; * *p* < 0.05 is considered statistically significant. *n*: number in each group; SLE: systemic lupus erythematosus; TNFSF4: Tumor necrosis factor superfamily member 4; IKZF1: IKAROS family zinc finger 1; ANA: anti-nuclear Antibodies; Anti-ds-DNA: anti-double-stranded DNA; WBCs: white blood cells; C3 & C4: complement components; S: serum Test; Logistic regression analysis; Chi-Square test; Kruskal–Wallis test; Mann–Whitney; One-Way ANOVA test.

**Table 4:** Association of TNFSF4 rs1234315 C/T, and IKZF1 rs11980379 C/T Genotypes with SLE Clinical Features.

Parameters		TNFSF4 rs1234315 C/T			*p*	IKZF1 rs11980379 C/T			*p*
		CC (*n* = 24)	CT (*n* = 54)	TT (*n* = 32)		TT (*n* = 76)	TC (*n* = 31)	CC (*n* = 3)	
Consanguinity									
	None	13 (54.2%)	33 (61.1%)	23 (71.9%)	0.376	46 (60.5%)	21 (67.7%)	2 (66.7%)	0.781
	Present	11 (45.8%)	21 (38.9%)	9 (28.1%)		30 (39.5%)	10 (32.3%)	1 (33.3%)	
Similar condition									
	None	17 (70.8%)	43 (79.6%)	22 (68.8%)	0.478	53 (69.7%)	26 (83.9%)	3 (100%)	0.227
	Present	7 (29.2%)	11 (20.4%)	10 (31.3%)		23 (30.3%)	5 (16.1%)	0 (0%)	
Lupus nephritis									
	Absent	4 (16.7%)	10 (18.5%)	9 (28.1%)	0.483	11 (14.5%)	9 (29%)	3 (100%)	0.002 *
	Present	20 (83.3%)	44 (81.5%)	23 (71.9%)		65 (85.5%)	22 (71%)	0 (0%)	
Fever									
	Absent	22 (91.7%)	48 (88.9%)	25 (78.1%)	0.294	68 (89.5%)	25 (80.6%)	2 (66.7%)	0.159
	Present	2 (8.3%)	6 (11.1%)	7 (21.9%)		8 (10.5%)	6 (19.4%)	1 (33.3%)	
Oral ulcer									
	Absent	18 (75%)	42 (77.8%)	25 (78.1%)	0.955	58 (76.3%)	25 (80.6%)	2 (66.7%)	0.664
	Present	6 (25%)	12 (22.2%)	7 (21.9%)		18 (23.7%)	6 (19.4%)	1 (33.3%)	
Vasculitis									
	Absent	21 (87.5%)	53 (98.1%)	31 (96.9%)	0.128	72 (94.7%)	30 (96.8%)	3 (100%)	1.0
	Present	3 (12.5%)	1 (1.9%)	1 (3.1%)		4 (5.3%)	1 (3.2%)	0 (0%)	
Arthritis									
	Absent	15 (62.5%)	29 (53.7%)	18 (56.3%)	0.770	43 (56.6%)	17 (54.8%)	2 (66.7%)	1.0
	Present	9 (37.5%)	25 (46.3%)	14 (43.8%)		33 (43.4%)	14 (45.2%)	1 (33.3%)	
Malar rash									
	Absent	11 (45.8%)	21 (38.9%)	13 (40.6%)	0.847	27 (35.5%)	16 (51.6%)	2 (66.7%)	0.201
	Present	13 (54.2%)	33 (61.1%)	19 (59.4%)		49 (64.5%)	15 (48.4%)	1 (33.3%)	
Photosensitivity									
	Absent	15 (62.5%)	31 (57.4%)	19 (59.4%)	0.914	46 (60.5%)	18 (58.1%)	1 (33.3%)	0.676
	Present	9 (37.5%)	23 (42.6%)	13 (40.6%)		30 (39.5%)	13 (41.9%)	2 (66.7%)	
Alopecia									
	Absent	16 (66.7%)	35 (64.8%)	20 (62.5%)	0.948	52 (68.4%)	18 (58.1%)	1 (33.3%)	0.254
	Present	8 (33.3%)	19 (35.2%)	12 (37.5%)		24 (31.6%)	13 (41.9%)	2 (66.7%)	
Neurological									
	Absent	18 (75%)	41 (75.9%)	24 (75%)	0.994	59 (77.6%)	22 (71%)	2 (66.7%)	0.552
	Present	6 (25%)	13 (24.1%)	8 (25%)		17 (22.4%)	9 (29%)	1 (33.3%)	
Duration		3 (0.5–10)	3 (1–10)	3.25 (0.5–9)	0.368	3.75 (0.5–10)	2 (0.6–7)	4 (3–10)	0.076
SLEDAI		10 (2–20)	7 (0–21)	10 (0–21)	0.325	9 (0–21)	10 (0–14)	4 (2–9)	0.451

Values are expressed as percentages; * *p* < 0.05 is considered statistically significant. *n*: number in each group; SLE: systemic lupus erythematosus; TNFSF4: Tumor necrosis factor superfamily member 4; IKZF1: IKAROS family zinc finger 1. Test, Logistic regression analysis, Chi-Square test, Kruskal–Wallis test, Monte Carlo test.

**Table 5:** Regression analysis for the prediction of different features using TNFSF4 rs1234315 C/T, and IKZF1 rs11980379 C/T dominant models among SLE.

Parameters	TNFSF4 rs1234315 C/T		IKZF1 rs11980379 C/T	
	*p*	OR (95% CI)	*p*	OR (95% CI)
SLEDAI	0.191	0.97 (0.92–1.02)	0.573	0.99 (0.94–1.04)
Renal biopsy	0.427	1.12 (0.84–1.50)	0.015 *	0.69 (0.51–0.93)
Lupus nephritis	0.559	0.82 (0.42–1.60)	0.016 *	0.49 (0.27–0.88)
Fever	0.382	1.46 (0.63–3.40)	0.166	1.63 (0.82–3.24)
Oral ulcer	0.765	0.91 (0.49–1.69)	0.719	0.90 (0.50–1.62)
Vasculitis	0.061	0.33 (0.11–1.05)	0.583	0.70 (0.19–2.51)
Arthritis	0.492	1.21 (0.71–2.06)	0.946	1.02 (0.62–1.67)
Malar rash	0.580	1.16 (0.68–1.97)	0.088	0.65 (0.39–1.07)
Photosensitivity	0.700	1.11 (0.65–1.90)	0.648	1.12 (0.68–1.85)
Alopecia	0.806	1.07 (0.62–1.86)	0.207	1.39 (0.83–2.30)
Neurological	0.953	0.98 (0.54–1.80)	0.431	1.25 (0.72–2.19)

* *p* < 0.05 is considered statistically significant. SLE: systemic lupus erythematosus; TNFSF4: tumor necrosis factor superfamily member 4; IKZF1: IKAROS family zinc finger 1. OR, odds ratio; CI, confidence interval. Test, Logistic regression analysis.

**Table 6:** Comparative analysis of TNFSF4 rs1234315 C/T and IKZF1 rs11980379 C/T genes in SLE.

Author	Ethnicity	Study Type	Population		Association
			Case	Control	
TNFSF4 rs1234315 C/T Gene					
Chua et al. (2016) [17]	Chinese	Case–control Cohort 1	336	359	No association
	Malay	Case–control Cohort 2	109	114	
	Indian	Case–control Cohort 3	31	36	
Yang et al. (2009) [22]	Chinese	Case–control Cohort 1	320	1500	Risk
	Thai	Case–control Cohort 2	2880	2700	
Han et al. (2009) [23]	Chinese	Case–control Cohort 1	1047	1205	Risk
		Case–control Cohort 2	1643	5930	
		Case–control Cohort 3	1509	1120	
Xu et al. (2019) [24]	Chinese (Guangxi)	Case–control Cohort	294	282	Risk
Zhang et al. (2011) [25]	Chinese (Han)	Case–control Cohort	1344	4315	Risk
	Chinese	Case–control Cohort 1	336	359	No association
	Malay	Case–control Cohort 2	109	114	
	Indian	Case–control Cohort 3	31	36	
Attia et al., current study	Egyptian	Case–control Cohort	110	122	Risk
IKZF1 rs11980379 C/T gene					
Chen et al. (2020) [15]	Chinese	Case–control Cohort	400	676	Protective
Attia et al., current study	Egyptian	Case–control Cohort	110	122	Protective

SLE Systemic lupus erythematosus, TNFSF4, Tumor necrosis factor superfamily member 4, IKZF1: IKAROS family zinc finger 1.

## Data Availability

The data presented in this study are available upon request from the corresponding authors.

## Abbreviations

The following abbreviations are used in this manuscript:

JSLE	Juvenile systemic lupus erythematosus
SLE	systemic lupus erythematosus
LN	lupus nephritis
IBD	inflammatory bowel disease
TNFSF4	tumour necrosis factor, member of the superfamily 4
OX40L	tumour necrosis factor (ligand), member of the superfamily 4
IKZF1	Ikaros family zinc finger 1
IKAROS	zinc finger transcription factor of the Krüppel family member
IRB	Institutional Research Board
SLICC/ACR	Systemic Lupus International Collaborating Clinics/American College of Rheumatology
SDI	damaged index
ISN/RPS	International Society of Nephrology/Renal Pathology Society
EDTA	Ethylene diamine tetra-acetic acid
OR	odds ratio
CI	confidence interval
WBCs	White blood cells
Ca	Calcium
P	Phosphorus
C3 and C4	Complements
ANA	Antinuclear antibodies
Ds-DNA	double-stranded DNA
